# Conjugated Polymer Nanoparticles for Label‐Free and Bioconjugate‐Recognized DNA Sensing in Serum

**DOI:** 10.1002/advs.201400009

**Published:** 2015-02-19

**Authors:** Biqing Bao, Mingfeng Ma, Huafeng Zai, Lei Zhang, Nina Fu, Wei Huang, Lianhui Wang

**Affiliations:** ^1^ Key Laboratory for Organic Electronics and Information Displays and Institute of Advanced MaterialsNanjing University of Posts and TelecommunicationsNanjing210023JiangsuP.R. China; ^2^ Key Laboratory of Flexible Electronics (KLOFE) and Institute of Advanced Materials (IAM)Jiangsu National Synergetic Innovation Center for Advanced Materials (SICAM)Nanjing Tech UniversityNanjing211816JiangsuP.R. China

**Keywords:** polyfluorene, nanoparticles, label‐free, DNA sensing

## Abstract

Hybridbio/‐synthetic sensory conjugated polymer nanoparticles (CPNs) are developed for selective label‐free detection of target ssDNA in serum. Carboxylic acid‐functionalized anionic polyfluorene nanoparticles are rationally designed as signal amplifying unit to bioconjugate with amine functionalized single stranded oligonucleotides as a receptor. The covalent DNA coating can signiﬁcantly improve the photostability of the DNA‐bioconjugated CPNs over a wide range of buffer conditions. Better ssDNA discrimination for the DNA‐bioconjugated CPNs sensor is achieved owing to increased interchain interactions and more efficient exciton transport in nanoparticles. The distinguishable ﬂuorescent color for DNA‐bioconjugated CPNs in the presence of target ssDNA allows naked‐eye detection of ssDNA under UV irradiation.

## 1 Introduction

Facile and reliable methods for detection of DNA are of vital importance to medical diagnosis, mutational analysis, gene therapy, biological studies, and specific genomic techniques.^1^, ^2^, ^3^ The innovative assays for DNA detection by conjugated polyelectrolytes (CPEs) have been reported recently, which combine the unique optical amplification properties of π‐conjugated polymers and the electrostatic behaviors of polyelectrolytes.^4^, ^5^, ^6^ Because CPEs contain a large number of absorbing units along the polymer backbone and can increase the probability of exiton energy transfer to fluorophore reporters, they provide a new platform for the detection of various analytes, such as metal ions, small biomolecules, DNA, and proteins.^7^, ^8^, ^9^, ^10^, ^11^ Label‐free detection of target biomolecules based on fluorescence resonance energy transfer (FRET) from CPEs to intercalating dyes is of great interest due to the simplicity and reduced cost. Intercalating dyes, such as ethidium bromide (EB),^12^ thiazole orange (TO),^13^ and Picogreen^14^ can act as energy acceptors in a CPE‐based biosensor and can achieve high sensitivity and simplicity. The sensing mechanism relies on electrostatic interactions between the anionic DNA and the cationic CPEs. When the dyes are intercalated within the anionic DNA hybrid, electrostatic interactions bring CPEs and dyes close, thus the fluorescence of CPEs can be transferred efficiently to dyes via the FRET process.

The limitation in this method is the requirement of the formation of the stable polyelectrolyte complexes between cationic CPEs and negatively charged DNA by electrostatic interactions,^13^, ^14^, ^15^ which have obvious background signals and give rise to false positives due to non‐specific interactions between CPEs and biomolecules. Generally, the challenges in using selective CPEs for label‐free DNA detection mainly arise from the ubiquitous non‐specific electrostatic and biological interactions between the polyelectrolytes and a large amount of biomolecules. The design of conjugated polymer sensors with precise optical signals toward label‐free DNA hence remains challenging.

Conjugated polymer nanoparticles (CPNs) represent a new class of fluorescent probes with superior characteristics, such as high fluorescence brightness, fast emission rates, and lower toxicity.^16^, ^17^, ^18^ Besides the extensive applications in cellular imaging and immunofluorescent labeling,^19^, ^20^, ^21^, ^22^ CPNs have also been verified to be a promising fluorescent probe for metal ion detection,^23^ intracellular pH value,^24^ and temperature sensing.^25^ Among these various existing applications, few CPNs have been used in DNA‐related sensors, thus seriously restricting the comprehensive applications of the CPNs. To address the need for facile and specific DNA sensing, further surface functionalization and subsequent bioconjugation of CPNs are needed to make the CPNs more practical in label‐free DNA‐related assays. To date, several methods have been used to modify the surface of CPNs, such as phospholipid encapsulation,^26^ amphiphilic polymer co‐condensation,^27^ and surfactant mini‐emulsion.^28^ However, the functional macromolecules are likely to dissociate from the formed CPNs due to the relatively weak non‐covalent interactions.

In this work, we report a new and simple bioconjugate‐recognized DNA sensor based on anionic carboxylic acid‐functionalized polyfluorene nanoparticles. The advantages of this design is that it does not utilize the electrostatic attraction of CPEs to detect anionic DNA and the CPNs sensors can improve detection sensitivity due to increased interchain interactions and more efficient exciton transport in nanoparticles. The covalent DNA coating can also significantly improves the photostability of the DNA‐bioconjugated CPNs over a wide range of buffer conditions, which is desirable for many applications and allows CPNs to be used under physiologically relevant environments. The further significance of this sensing system is that intercalating dye Picogreen are used in combination with the DNA‐bioconjugated CPNs to develop label‐free DNA sensors and thus a detection concentration as low as nanomolar could be achieved in serum.

## 2 Results and Discussion

### 2.1 Preparation and Characterization of PF‐COOH CPNs

**Scheme 1:**
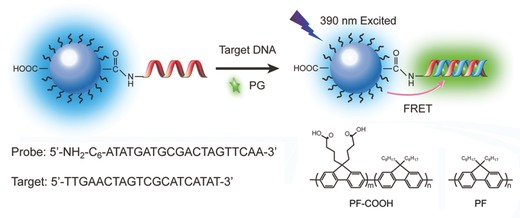
Schematic illustration of the PF‐DNA_P_ CPNs for label‐free DNA detection.

**Figure 1:**
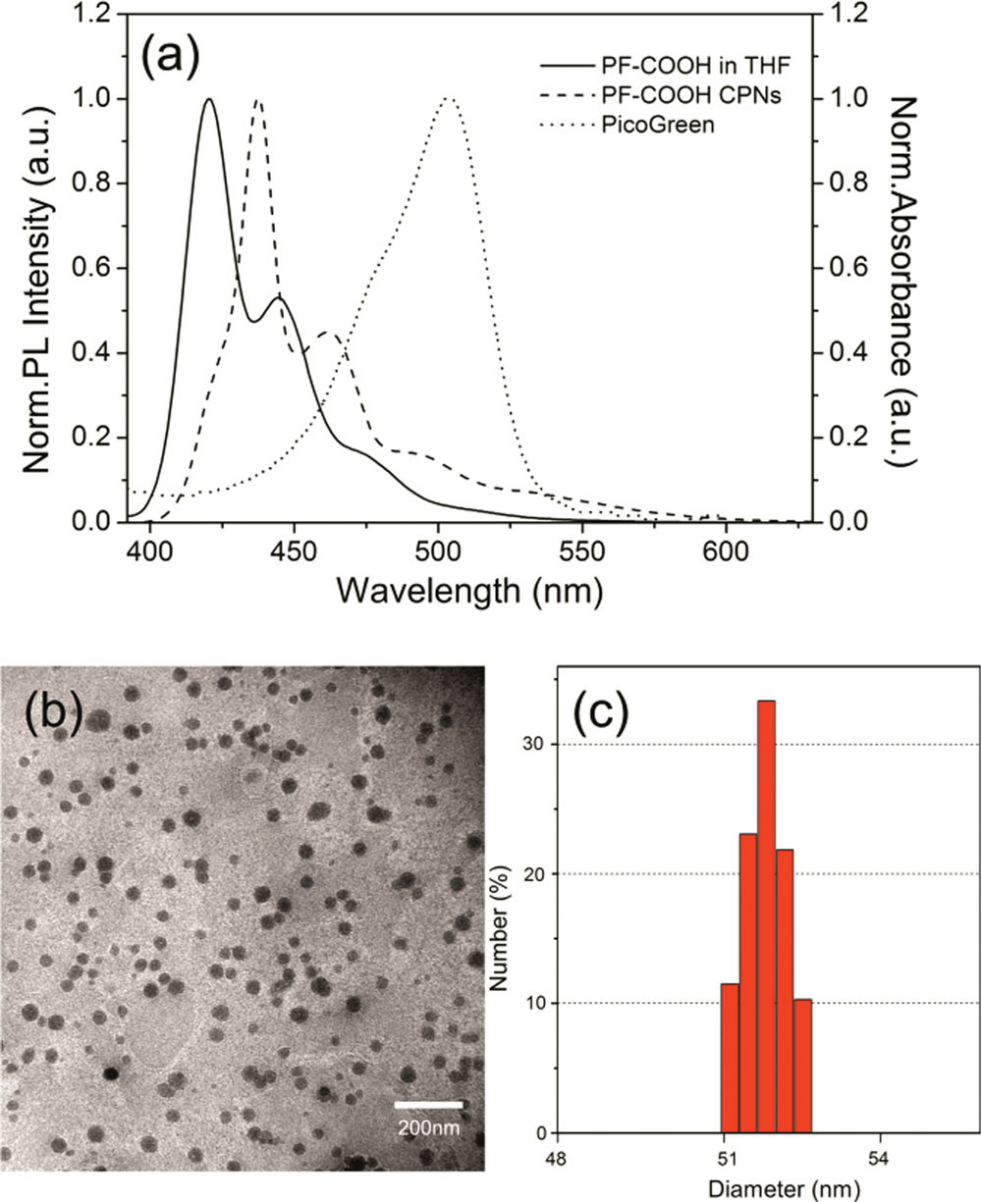
a) Emission spectra of PF‐COOH in THF, PF‐COOH CPNs in aqueous solution and absorption spectrum of PG. b) Transmission‐electron‐microscopy for PF‐COOH CPNs. c) Dynamic‐light‐scattering measurements of PF‐COOH CPNs.

To overcome non‐specific biological interactions between the polycationic conjugated polymers and charged biomolecules, the anionic carboxylic acid‐functionalized polyfluorenes (PF‐COOH, **Scheme** **1**) were employed to form PF‐COOH CPNs by a reprecipitation procedure. The PL spectra of PF‐COOH in THF and PF‐COOH CPNs in aqueous solution are shown in **Figure** **1**a. The as prepared PF‐COOH CPNs exhibits red‐shifted fluorescence spectra as compared to its corresponding polymer in THF solution, which is similar with previous literatures and is caused by increased interchain interactions in nanoparticles.^29^ Figure 1a also shows the increased overlap between the emission of PF‐COOH CPNs and the absorption of PicoGreen (PG) in the 400–550 nm range, which should make more efficient FRET from PF‐COOH CPNs to PG. And we also can expect that the exciton transport of CPEs in solution is less sensitive than CPNs due to the more densely packed structure and increased interchain interactions in nanoparticles.^30^, ^31^ To investigate their particle size and morphologies, the PF‐COOH CPNs were characterized by both TEM and DLS as shown in Figure 1b,c. The particle size obtained from DLS indicated that the majority of PF‐COOH CPNs possessed hydrodynamic diameters in the range of ca. 51 nm (Figure 1c).

### 2.2 PF‐COOH CPNs and DNA Bioconjugation

**Figure 2:**
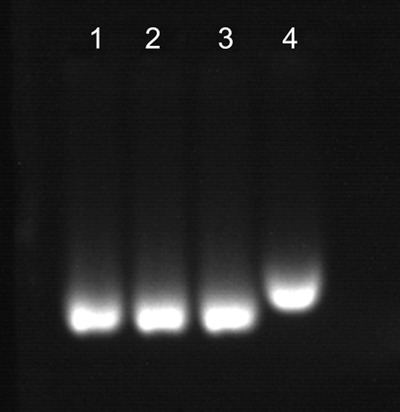
Gel electrophoresis of bare PF‐COOH CPNs (lane 1), PF‐COOH CPNs mixed with EDC (lane 2), PF‐COOH CPNs mixed with DNA_P_ (lane 3), and PF‐DNA_P_ CPNs(lane 4).

To apply PF‐COOH CPNs for specific DNA detection, we successfully performed bioconjugation of oligonucleotides to the functionalized PF‐COOH CPNs. The oligonucleotide probe used was 5′‐NH_2_‐C_6_‐ATATGATGCGACTAGTTCAA‐3′ (DNA_P_‐NH_2_), with an amine group at the 5 position. As illustrated in Scheme 1, amino‐modified oligonucleotides were attached to –COOH groups on the surfaces of PF‐COOH CPNs via conventional bioconjugation chemistry using 1‐ethyl‐3‐(3‐dimethylaminopropyl)carbodiimide hydrochloride (EDC) catalyst. An excess amount of the oligonucleotides were added to the PF‐COOH CPNs solution to ensure effective binding of the oligonucleotides to the nanoparticles. Bioconjugation between oligonucleotides and the functional groups on the PF‐COOH CPNs surface was confirmed by agarose gel electrophoresis (0.5%). **Figure** **2** shows that PF‐DNA_P_ CPNs (lane 4) exhibit an apparent decrease in mobility compared to bare PF‐COOH CPNs (lane 1) in the agarose gel. PF‐COOH CPNs treated with aminated DNA_P_‐NH_2_ and PF‐COOH CPNs treated directly with EDC in the absence of DNA_P_‐NH_2_ were also used as control as shown in lane 2 and lane 3. These results indicate the successful surface bioconjugation of PF‐COOH CPNs with oligonucleotides since the DNA bioconjugated nanoparticles have slower migration mobility compared to bare PF‐COOH CPNs due to the slightly larger molecular weight of PF‐DNA_P_ CPNs.

### 2.3 Photostability of PF‐DNA_P_ CPNs

**Figure 3:**
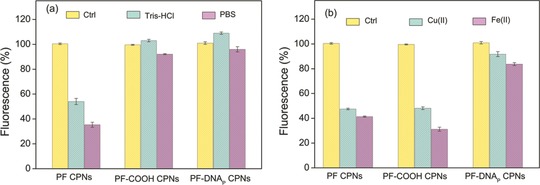
Changes in fluorescence intensity of bare PF CPNs, PF‐COOH CPNs and PF‐DNA_P_ CPNs in a) Tris‐HCl (pH 7.4), PBS (pH 7.4), and b) in 0.5 mm Cu(II) (CuSO_4_ in DI water) and 0.4 mm Fe(II) (FeSO_4_ in DI water). Control samples were dispersed in DI water.

To demonstrate the nanoparticle stability of PF‐DNA_P_ CPNs, we measured the intensity of fluorescence emission from PF CPNs, PF‐COOH CPNs and PF‐DNA_P_ CPNs since the emission efficiency of CPNs is critically important for many fluorescence‐based biological applications and it is also a sensitive way to monitor nanoparticle stability.^32^ **Figure** **3**a shows the fluorescence intensities of PF CPNs, PF‐COOH CPNs and PF‐DNA_P_ CPNs dispersed in PBS and Tris‐HCl buffer solution. The fluorescence intensity of bare PF CPNs showed nearly 47% reduction in Tris‐HCl and up to 66% fluorescence quenching in PBS solution. On the contrary, PF‐COOH CPNs and PF‐DNA_P_ CPNs displayed significant improvement of optical stability and do not show obvious fluorescence change in various buffers such as PBS and Tris‐HCl. For evaluating the effect of different ions on the optical stability of PF‐DNA_P_ CPNs, the PF‐DNA_P_ CPNs solution were also titrated with different metal ions such as 0.4 mm Fe(II) solution, 0.5 mm Cu(II) solution, 0.5 mm Ca(II) solution, and 0.5 mm Mg(II) solution. As shown in Figure 3b, the addition of Fe(II) and Cu(II) both leads to effective quenching of the PF CPNs and PF‐COOH CPNs. The mechanisms of Cu(II) and Fe(II)‐induced fluorescence quenching of bare PF CPNs and PF‐COOH CPNs may be due to the screened charge repulsion of nanoparticles by the increase in ionic strength and the specific interactions between CPNs and the biovalent metal ions.^32^, ^33^ Fluorescence measurements also shows no obvious change of fluorescence intensity for PF‐DNA_P_ CPNs in the presence of Cu(II) and Fe(II) solutions, which may be due to the combination of charge and steric stabilization by DNA coating. These results demonstrate that DNA functionalization can be applied to achieve high optical stability of CPNs in various buffer solutions, metal ions for many biological applications.

### 2.4 Fluorescence Response toward DNA

**Figure 4:**
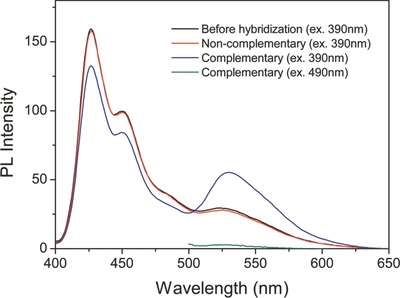
PL change of PF‐DNA_P_ CPNs/PG before and after hybridization between complementary target ssDNA_C_ (excitation at 390 nm and 490 nm) and non‐complementary ssDNA_NC_. [ssDNA_C_] = [ssDNA_NC_] = 8 × 10^−9^ m; λ_ex_ = 390 nm.

The DNA detection is illustrated in Scheme 1, PF‐DNA_P_ CPNs is used as the energy donor and PicoGreen (PG) as the DNA intercalator and energy acceptor. Results for the detection of target ssDNA_C_ using PF‐DNA_P_ CPNs probe are shown in **Figure** **4**. The solution of PF‐DNA_P_ CPNs/PG exhibits a strong emission at 426 nm and do not show obvious change after hybridization with non‐complementary DNA(ssDNA_NC_), when the PF‐DNA_P_ CPNs/PG/DNA_NC_ complex was selectively excited under excitation of PF‐DNA_P_ CPNs at 390 nm. After hybridization with target ssDNA_C_, the blue emission band of PF‐DNA_P_ CPNs at 426 nm displays fluorescence quenching along with the obvious growth of green emission band at 530 nm. The results suggest that FRET is not observed in the presence of ssDNA_NC_ and a fairly good selectivity can be achieved between complementary and noncomplementary ssDNA when PF‐DNA_P_ CPNs was used as the fluorescence probe. Furthermore, the fluorescence intensity of PG of the PF‐DNA_P_ CPNs/PG/DNA_C_ complex ([ssDNA_C_] = 8 × 10^−9^ M) was amplified more than 19 times compared to the emission intensity when PG was directly excited at the absorption maxima of PG at 490 nm as shown in Figure 4. This fluorescence enhancement implies further improved sensitivity for DNA detection by using DNA bioconjugated CPNs probe than conventional conjugated polyelectrolyte‐based DNA sensors.

**Figure 5:**
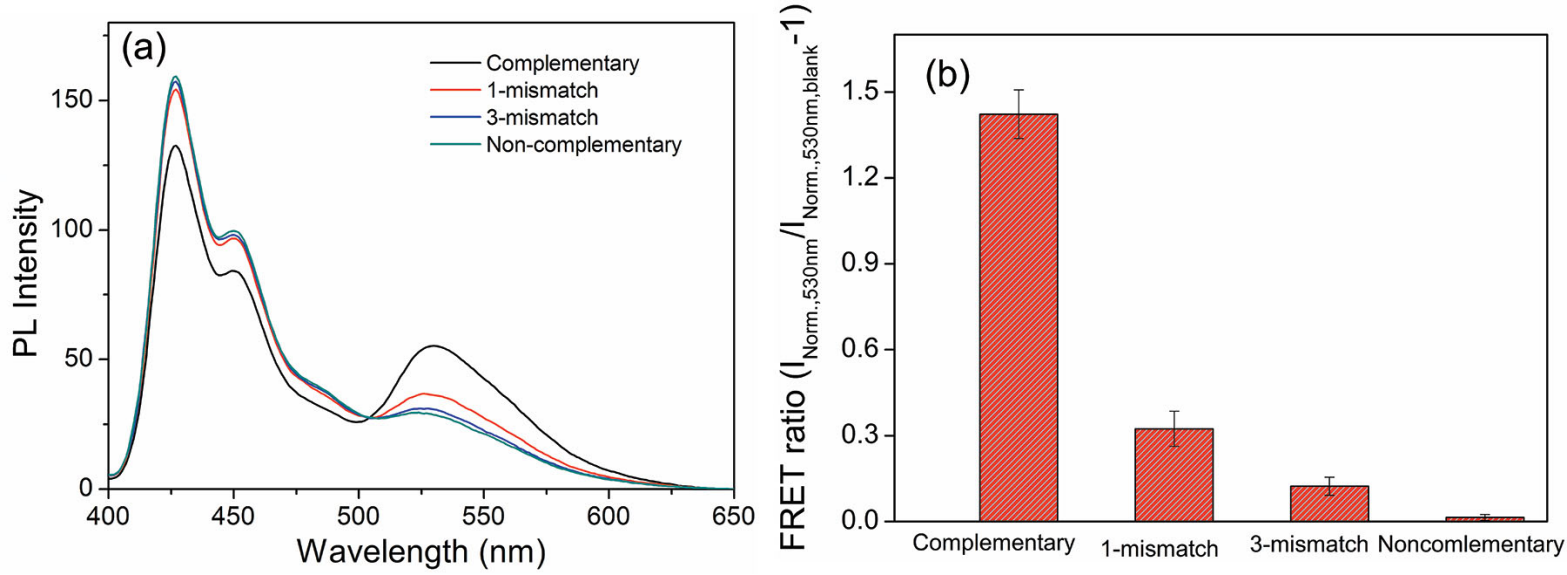
a) PL spectra of PF‐DNA_P_ CPNs/PG/DNA with increasing number of mismatched base pairs and b) Normalized emission intensity of PF‐DNA_P_ CPNs/PG/DNA with increasing number of mismatched base versus blank PF‐DNA_P_ CPNs/PG at 530 nm (I_Norm.,530 nm_/I_Norm.,530 nm,blank_‐1). [ssDNA_C_] = [ssDNA_1NC_] = [ssDNA_3NC_] = [ssDNA_NC_] = 8 × 10^−9^ m; λ_ex_ = 390 nm.

To further explore the improved selectivity of PF‐DNA_P_ CPNs for DNA detection, a series of control experiments were performed by using single‐mismatched and 3‐mismatched ssDNA. **Figure** **5** shows the emission spectra of PF‐DNA_P_ CPNs/PG/DNA with an increasing number of mismatched base pairs at room temperature. The hybridization of PF‐DNA_P_ CPNs with fully complementary ssDNA_C_ showed efficient energy transfer and the emission spectrum of the complexes with one base pair mismatch is significantly different from that of the fully complementary case. Furthermore, the energy transfer efficiency decreased with an increasing number of mismatched base pairs in DNA strands. The results show that single base pair mismatch could be easily detected even at room temperature and PF‐DNA_P_ CPNs could be used to improve both the sensitivity and selectivity.

### 2.5 Recognition of ssDNA in Serum

**Figure 6:**
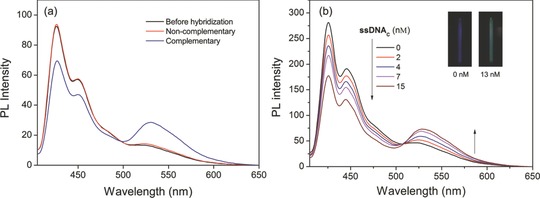
a) PL change of PF‐DNA_P_ CPNs/PG before and after hybridization between complementary target ssDNA_C_ and non‐complementary ssDNA_NC_ in HEPES containing 10 vol% serum. [ssDNA_C_] = [ssDNA_NC_] = 8 × 10^−9^ m. b) PL spectra of PF‐DNA_P_ CPNs/PG in the presence of ssDNA_C_ with [ssDNA_C_] ranging from 0 to 15 nm in HEPES containing 10 vol% serum. Insets: fluorescence photographs of PF‐DNA_P_ CPNs/PG with and without ssDNA_C_. λ_ex_ = 390 nm.

As reported, many conjugated polyelectrolyte‐based biosensors rely on the non‐specific electrostatic interactions between cationic conjugated polymers and anionic nucleotides. The detection specificity is subject to interferences from other biological and nonbiological macromolecules due to wide‐ranging non‐specific electrostatic interactions between conjugated polyelectrolytes and other macromolecules, which indicate that biological recognition is necessary for the detection of biological macromolecules. To demonstrate that PF‐DNA_P_ CPNs is able to recognize target ssDNA_C_ in biological media, DNA hybridization and the measurement experiments were carried out in HEPES buffer (PH = 7.4) containing 10 vol% serum. **Figure** **6**a shows the fluorescence response of PF‐DNA_P_ CPNs toward ssDNA_C_ and ssDNA_NC_ in the serum containing buffer at [DNA] = 8 nm. The presence of ssDNA_NC_ in the PF‐DNA_P_ CPNs/PG solution does not increase the PG emission and the energy transfer in the presence of ssDNA_C_ is substantially higher than that in the presence of ssDNA_NC_, which indicates that PF‐DNA_P_ CPNs is capable of distinguishing ssDNA_C_ in biological media.

The FRET efficiency of PF‐DNA_P_ CPNs/PG in serum with different ssDNA_C_ concentrations (0–15 nm) after excited at 390 nm was showed in Figure 6b. It is obvious that additon of ssDNA_C_ can lead to obvious emission intensity increment at 530 nm. The results also show that a detection concentration as low as nanomolar for target ssDNA_C_ could be achieved in serum containing solution, which was better than or comparable with those of existing CPEs‐based homogeneous assays. The color change of the emission fluorescence from PF‐DNA_P_ CPNs solution with ssDNA_C_ can also be monitored by the naked eye under UV irradiation. As shown in the insets of Figure 6b, the fluorescent color of PF‐DNA_P_ CPNs/PG solution is blue in the absence of ssDNA_C_, while it changes to light cyan with increasing [ssDNA_C_].

## 3 Conclusions

In summary, we demonstrate a simple platform that employs anionic DNA‐functionalized polyfluorene nanoparticles for label‐free oligonucleotides detection. Label‐free DNA hybridization assays based on conjugated polyelectrolytes and DNA interclators are attractive because of their simplicity of operation and use of standard optical equipment. However, the energy transfer efficiency was limited and the selectivity of conjugated polyelectrolytes‐based biosensors is usually not satisfactory due to the non‐specific interactions between conjugated polyelectrolytes and biomacromolecules. The detection of oligonucleotides in complex media and discrimination of single nucleotide mismatch is limited. To improve both the sensitivity and selectivity, here hybrid biological sensory conjugated polymer nanoparticles are developed to achieve selective and label‐free detection of target oligonucleotides in biological media. The bioconjugate‐recognized CPNs sensor is able to detect as low as nanomolar target ssDNA_C_, which is significantly sensitive than common DNA sensor based on conjugated polyelectrolytes. Furthermore, the DNA functionalization dramatically improves the photostability of the CPNs in buffer solutions and allows CPNs to be used under physiologically relevant environments and harsh conditions. This simple, sensitive, and economical CPNs design could also be generalized for the detection of other chemical and biological substances simply by choosing suitable biomolecules as the recognition element.

## 4 Experimental Section

*Monomer Synthesis*: 2,7‐Dibromo‐9,9‐dioctylfluorene (**M1**)^34^ and 2,7‐bis(4,4,5,5‐tetramethyl‐1,3,2‐dioxaborolan‐2‐yl)‐9,9‐bis(4‐ethylbutyrate)fluorene (**M2**)^35^ were prepared according to the literature methods.

*General Procedure for Synthesis of PF‐COOH*: The polymer PF‐COOC_2_H_5_ was synthesized by Suzuki coupling polymerization. **M1**, **M2** and Pd(PPh_3_)_4_ (2.0 mol%) were added to a 25‐mL flask. After degassed and charged with nitrogen, the degassed toluene and 2 M K_2_CO_3_ aqueous solution were added. The mixture was stirred at 80 °C under nitrogen atmosphere for 48 h, and then excess amount of bromobenzene was added and stirring continued for 12 h. After cooling to room temperature, the solution was concentrated and precipitated from methanol twice. The precipitate was filtered and Soxhlet extracted with methanol for 48 h. The carboxylate‐containing polymer PF‐COOC_2_H_5_ (200 mg) was dissolved in THF (80 mL) and treated with NaOH (1 g, 25 mmol). The mixture was refluxed for 12 h and acidied with Hydrogen chloride. The obtained solution was poured into ethanol and collected to give PF‐COOH as a dark greenish solid. **PF‐COOH**: ^1^H NMR (400 MHz, Tetrahydrofuran‐d_8_): d (ppm) 10.86 (s, 2H, COOH), 8.03–7.47 (m, 12H, ArH), 2.74‐2.39 (br, 4H, CH_2_), 2.35‐2.13 (br, 4H, CH_2_), 2.12–1.87 (br, 4H, CH_2_), 0.51–1.38 (br, 34H). The molecular weight of PF‐COOC_2_H_5_ was measured by GPC as Mn = 36809, Mw = 69037, PDI = 1.875.

*Preparation and DNA Bioconjugation of PF‐COOH CPNs*: PF‐COOH CPNs in aqueous solution were prepared by a reprecipitation method. In a typical preparation, PF‐COOH was first dissolved in tetrahydrofuran (THF) to make a 0.1 mg mL^−1^ stock solution. 2‐mL quantity of THF solution of PF‐COOH was then quickly added to 8 mL MilliQ water in a vigorous bath sonicator. The THF was removed by partial vacuum evaporation, followed by filtration through a 0.22 μm filter. All reagents were immediately handled and used before bioconjugation. Bioconjugation between PF‐COOH CPNs and amino‐functionalized ssDNA_P_ was conducted by standard carbodiimde chemistry. To a solution of PF‐COOH CPNs and amino‐functionalized 20‐base DNA in MES buffer (pH = 5.5) solution, EDC was directly added and the reaction mixture was stirred for 5 h in a dark room at room temperature. Unbound oligonucleotides from the PF‐DNA_P_ CPNs solution were removed by centrifugal washing with DI water several times using microcentrifuge tube (MWCO = 1 000 000) until no change in characteristic UV absorbance (260 nm) from the filtrate solution was observed (Figure S1, Supporting Information).

## Supporting information

As a service to our authors and readers, this journal provides supporting information supplied by the authors. Such materials are peer reviewed and may be re‐organized for online delivery, but are not copy‐edited or typeset. Technical support issues arising from supporting information (other than missing files) should be addressed to the authors.

Supplementary
