# Supplementary material for: Conjugated Polymer Nanoparticles for Label‐Free and Bioconjugate‐Recognized DNA Sensing in Serum
Source: Adv Sci (Weinh). 2015 Feb 19;2(3):1400009. doi: 10.1002/advs.201400009 (PMC5024028; doi:10.1002/advs.201400009)
Supplement: Supplementary file 1 — Supplementary [file ADVS-2-0a-s001.pdf]

Copyright WILEY-VCH Verlag GmbH & Co. KGaA, 69469 Weinheim, Germany, 2015.

ADVANCED SCIENCE
Open Access

Supporting Information

for *Adv. Sci.*, DOI: 10.1002/advs. 201400009

Conjugated Polymer Nanoparticles for Label-Free and Bioconjugate-Recognized DNA Sensing in Serum

Biqing Bao, Mingfeng Ma, Huafeng Zai, Lei Zhang, Nina Fu, Wei Huang,* and Lianhui Wang*

Semiconducting Polymer Nanoparticles for Label-Free and Bioconjugate-Recognized DNA Sensing in Serum

Biqing Bao[†] *Mingfeng Ma*[†] *Huafeng Zai*[†] *Lei Zhang*[†] *Nina Fu*[†] *Wei Huang*[*,‡] *Lianhui Wang*[*,†]

†Key Laboratory for Organic Electronics and Information Displays and Institute of Advanced Materials,Nanjing University of Posts and Telecommunication, Nanjing 210023, China

‡Key Laboratory of Flexible Electronics (KLOFE) & Institute of Advanced Materials (IAM), National Synergistic Innovation Center for Advanced Materials (SICAM), Nanjing Tech University, Nanjing 211816, China

Materials

All starting materials and reagents for monomers and polymer preparation were used without further purification as purchased from Sigma-Aldrich Chemical Co. and Aladdin reagent (Shanghai, China). Distilled toluene was used for the preparation of PF-COOH according to standard procedures.1-ethyl-3-(3-dimethylaminopropyl) carbodiimide hydrochloride (EDC) and 2-(N-Morpholino)ethanesulfonic acid (MES) were purchased from Aladdin reagent for bioconjugation. All oligonucleotides as well as bovine serum albumin were purchased from Sangon Biotechnology Co., Ltd. (Shanghai, China) and used without further purification. Concentrations of the oligonucleotides were determined by measuring their absorbance at 260 nm. MilliQ water was used to prepare all stock solutions used in the experiments.

Characterization

The color changes in PL spectra were measured using a Shimadzu RF-5301PC spectrophotometer. UV-vis spectra were acquired on a Shimadzu UV-3600PC UV-visible scanning spectrophotometer at room temperature. The NMR spectrum was recorded on a Bruker AV 400 MHz NMR spectrometer. The gel permeation chromatography (GPC) analysis was conducted at room temperature on a Shim-pack GPC-80X column using polystyrene as a standard and tetrahydrofuran (THF) as the eluant. The size and size distribution of CPNs in aqueous solution were measured by dynamic light scattering (DLS) and carried out on a Brukehaven ZetaPALS with a He-Ne laser (633 nm) and 90 ^{0}C collecting optics. Transmission electron microscopy (TEM) was performed on a Hitachi HT7700 operating at 100 kV accelerating voltage.

Supporting Figures

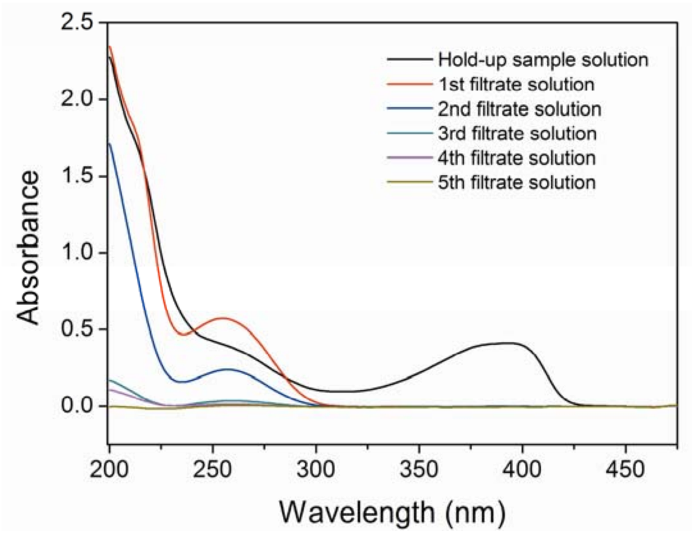

Supplementary Figure S1 UV-visible absorption spectra of hold-up PF-DNA_P CPNs solution and filtrate solution by centrifugal washing.
